# Decreasing consumption of sugar-sweetened beverages and Raising tap water consumption through Interventions based on Nutrition and sustainability for Kids: study protocol of the “DRINK” cluster randomised controlled trial

**DOI:** 10.1186/s13063-023-07643-z

**Published:** 2023-09-26

**Authors:** Katia Castetbon, Wassila Assakali, Isabelle Thiébaut, Lucille Desbouys

**Affiliations:** 1 Research Center in “Epidemiology, Biostatistics and Clinical Research”, School of Public Health, Université libre de Bruxelles (ULB), CP598, Route de Lennik 808, Brussels, 1070 Belgium; 2 Club Européen des Diététiciens de L’Enfance (CEDE), Esplanade 17, Ath, 7800 Belgium

**Keywords:** Beverages, Children, Diet surveys, Drinking water, Environment and public health, Health promotion, Primary schools, Randomised controlled trial, Sugar-sweetened beverage

## Abstract

**Background:**

Effectiveness of actions to reduce sugar-sweetened beverage (SB) consumption in children still needs to be improved. Furthermore, the growing concern about sustainable food systems encourages to develop sustainability-based interventions. The objective of this cluster randomised controlled trial is to evaluate the long-term effectiveness of nutrition- and environmental sustainability-based interventions on the reduction in SB intake and on the increase in tap water consumption in 3rd to 6th grade primary school children (8 to 11 years of age).

**Methods:**

Forty-eight French-speaking Belgian primary schools (equivalent to around 3500 pupils involved in the evaluation) are randomised using a factorial plan: (i) control, (ii) nutrition-based intervention, (iii) sustainability-based intervention, and (iv) both. The interventions (encouragement of water breaks; provision of posters, leaflets, reusable cups, and glass bottles; website; meetings at school) were undertaken from February 2022 to June 2023. Evaluation includes questionnaires for the children and their parents on various determinants of dietary behaviour, a 4-day diary to collect information on the child’s beverage consumption, and audits at schools. The first evaluation was conducted in Spring 2021 before any intervention, with the two post-intervention evaluations being held in 2022 and 2023. The main quantitative judgement criterion will be the change over time in the mean SB consumption (in ml/day) in the intervention groups compared with the control group. Given the context of the research (school), the safety of the intervention, and the content of data collection, a consent was acknowledged as unnecessary by the Ethical Committee of the Faculty of Psychology (ULB; n°073/2021), but children and parents are explicitly informed of their right to refuse to fill in the questionnaires.

**Discussion:**

Multicomponent interventions based on nutrition and on environmental sustainability, alone or mixed, will provide an original and topical insight into health promotion at school around dietary behaviours. The dissemination plan will enable to widely inform stakeholders, school staff, and families, in addition to the scientific community through the usual medium (articles, conferences), about the research findings in 2024–2025.

**Trial registration:**

ISRCTN Registry ISRCTN99843102. Retrospectively registered on 25 May 2021

## Introduction

### Background and rationale {6a}

For several decades, regular sugar-sweetened beverage consumption in children is one of the most common unhealthy dietary habits worldwide [1]. Its association with weight gain and obesity and the risk of dental caries has been clearly documented and underlined as particularly worrisome [1]. Despite declines in most high-income countries, levels of consumption remain too high overall [2]. Substitution to water consumption, and not to artificially sweetened beverages (also included in sweetened beverages (SB)), is a topical issue to decrease sugar-liking. Furthermore, overall hydration is often considered as insufficient [3]. The consequences of chronic inadequate water intake on health are much less clearly established [4, 5]. In particular, it may affect attention and cognitive performance in children [6]. Therefore, reducing SB consumption should go in pair with the increase in drinking plain water, especially since substitution is not systematic [7].

To reduce SB beverage consumption, especially among young people, some public health initiatives like taxes, advertising regulation, and reduced exposure in children’s usual places of living have been developed in some countries [8]. In addition, schools are potential settings where such an issue can be effectively addressed. Indeed, they are considered as relevant intervention places: they are supposed to be healthy environments, and health education is included in teaching [9]. However, SB are still available in some schools via vending machines or easily accessible around schools [10]. In addition, children may come to school with snacks including SB, due to their easy and pleasant use, but also in relation with a poor or restricted access to tap water at school [11]. Beyond the environment offered by the school, increasing awareness and involving parents at some point in such a behavioural change are crucial [12].

Several trials have been developed to improve beverage-related behaviour in primary school children [13–16]. They included various intervention contents (information, accessibility) and targets (children, parents, teachers—individually or together). Depending on the criteria of judgement, research has reported mixed findings [16]. Essentially, whereas decreased sugary-sweetened beverages and increased water consumption were reported most of the time [17–24], positive effects on body weight status were less likely [25, 26] although they have been shown in some trials [27, 28]. However, findings of most trials published so far have not been valued enough due to methodological reasons or unconvincing conclusions: short-term assessment, complex intervention package difficult to include in the daily life of school staff, uncontrolled or incorrectly randomised trials, particular populations, etc. [13, 15, 16]. Strikingly, this topic is subject to conflicts of interest which makes it difficult to disentangle study limits and the influence of private interests [29]. Thus, undertaking trials independently of funding from beverage producers is crucial.

More recently, a large body of evidence has been collected on the relationship between foods, dietary habits, and the environment [30]. Dietary habits, i.e. the food groups consumed, are particularly impactful on various environmental indicators [30, 31]. Along with the concern in the general population around the COVID-19 origins and climate change impact, demonstrations led by the young Europeans (e.g. the “Youth for Climate” initiative) encourage to evaluate whether new opportunities for health promotion can be addressed in this way. So far, very limited investigation has been made to appraise how environment-related messages would help modify behaviours [32, 33]. Indeed, substituting SB with tap water reduces the production, cost, and waste impact in addition to its benefits on health. Still, the effectiveness of basing interventions on such issues, along with the changes in the school environment (e.g. facilitating access to tap water), must be evaluated. From a conceptual point of view, the underlining cognitive process would differ from the one involved in nutrition-based arguments [34]. In this case, the expected benefits are collective, rather than individual as for health-related changes, and in the long term if ever measurable over a lifetime. Yet, the theory of planned behaviour (TPB) that may support the identification of potential mediating factors in nutrition and environmental behaviour change suggests a successful intervention basis [35, 36]. Also, emotional, oral and normative concerns related to the environment call for an extended conceptual framework of analysis [36].

In Belgium, SB consumption is particularly high in the general population including children [37], putting this country in the top five of the European countries with the highest prevalence of daily consumers [38]. After a decrease until the early 2000s, a plateau in daily consumption has been reported since [2]. In addition, social inequalities in SB consumption tend to increase in Belgian adolescents [39]. Furthermore, the overall water intake is considered insufficient in Belgium [40, 41] like elsewhere [42, 43].

Based on the SPIRIT checklist [44], this paper describes the protocol of the DRINK trial, for “Decreasing consumption of sugar-sweetened beverages and Raising tap water consumption through Interventions based on Nutrition and sustainability for Kids”, a cluster randomised controlled trial (cRCT) implemented in Belgian French-speaking schools. The originality of the DRINK trial lies in studying the potential interacting effectiveness of interventions with enablers in both nutrition and sustainability, by using a factorial plan of randomisation.

### Objectives {7}

The first objective of the DRINK trial is to evaluate the long-term effectiveness of nutrition- and sustainability-based interventions on the reduction in SB intake and on the increase in tap water consumption in 3rd to 6th grade children (8 to 11 years of age).

The secondary objectives are (i) to specifically study the potential interaction between nutrition- and sustainability-based information provided within the interventions, (ii) to identify individual and family moderators of the intervention effectiveness, (iii) to assess school staff adherence to the set of interventions and to identify obstacles in the implementation of the interventions, and (iv) to estimate the total cost of the interventions in order to plan their generalisation.

### Trial design {8}

**Fig. 1:**
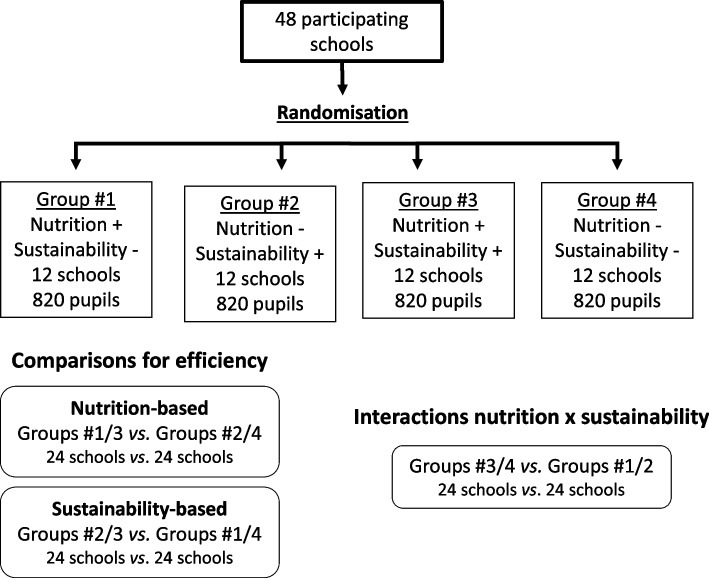
Factorial randomisation plan and planned analyses of the DRINK trial. Numbers correspond to the theoretical sample size needed (see the “Sample size {14}” section)

DRINK is a cluster randomised trial carried out from 2021/2022 to 2023/2024, where clusters are French-speaking primary schools in Belgium. The randomisation follows a factorial plan leading to allocate schools into four balanced groups (Fig. 1): (1) nutrition-based interventions, (2) sustainability-based interventions, (3) both nutrition- and sustainability-based interventions, and (4) control schools.

## Methods: participants, interventions, and outcomes

### Study setting {9}

The DRINK trial is conducted in the French-speaking schools of Belgium, which are geographically located in the Brussels-Capital Region and in Wallonia. Primary schools are organised in several networks (public and private), the education programmes being established by the Federation of Wallonia-Brussels (FWB).

### Eligibility criteria {10}

All schools under the aegis of the Ministry of Education (FWB) were eligible, the exception being those solely for children with disabilities or special needs. The design of the study requiring to follow students during two school years and for organisation reasons, schools not having students in 3rd to 6th grades, and those with less than 11 students by grade were excluded from the selection. Among the participating schools, all children schooled in the 3rd to 5th grades (8 to 10 years of age) at the initial evaluation were eligible unless they had important difficulties in reading and understanding French (such as some foreign children who have just arrived in Belgium).

### Who will take informed consent? {26a}

Given the context of the research (school), the intervention, and the content of data collection, a consent was not necessary. Instead, children and parents are informed of their right to refuse to complete the questionnaires. Refusal forms are kept at schools because all research data are kept pseudonymised to the research team.

### Additional consent provisions for collection and use of participant data and biological specimens {26b}

This trial does not involve collecting biological specimens for storage.

## Interventions

### Explanation for the choice of comparators {6b}

Since the randomisation follows a factorial plan, the comparators will include different control schools for each domain of intervention (Fig. 1). We will compare (1) groups #1 and #3 to groups #2 and #4 for assessing the effectiveness of nutrition-based actions, (2) groups #2 and #3 to groups #1 and #4 for sustainability-based actions, and (3) groups #3 and #4 to groups #1 and #2 for assessing any interaction between the two intervention types. Therefore, only schools allocated to group #4 will not benefit from the research intervention packages. However, the staff from these schools are encouraged to continue their ongoing projects about nutrition and sustainability, such situations being documented in the school questionnaires.

### Intervention description {11a}

**Fig. 2:**
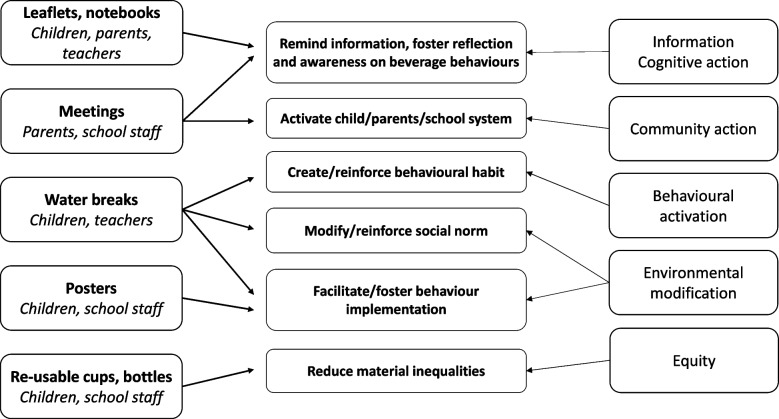
Intervention package and purposes of each component based on theory of planned behaviour (TPB) components. All documents are made available on a website, along with video capsules and interactive games for the children. Access to website is differentiated according to the group

The intervention package includes complementary actions aiming to promote various components potentially involved in health behaviours, namely the theory of planed behaviour [35, 36] (Fig. 2). Through the different actions, the interventions aim to include cognitive action (through documents and meetings), reinforce the community level (meetings), activate health behaviours (water breaks), and modify the school environment. Equity is addressed through the supply of bottles only to the children who need them. Such actions may act on attitudes, norms, and self-efficacy, these dimensions being documented in the questionnaires (see the “Plans for assessment and collection of outcomes {18a}” section).

The intervention package has been developed to be easily included in the daily life of schools, without important structural changes. Indeed, the school staff must deal with constraints in relation to the official educational programme changes and encouragement to include extra-curricular activities. Furthermore, the school staff are trained through various documents and regular phone contacts. In situ training has not been organised so that it can easily be generalisable to (all) the school authorities. To ensure long-term maintenance of the intervention, we purposely developed it in a way to be as adaptable as possible to various school situations.

In the three groups of intervention, some components are implemented towards either the school staff, children, or parents (Fig. 2 and Table 1). “Physical” actions (supply of bottles and reusable cups, water breaks, meetings) have been developed alongside information documents that disseminate general knowledge on the topics and practical suggestions (tips and answers to “frequently asked questions” adapted to each target (Table 1)). For instance, more information, such as scientific basis, is provided in teachers’ documents compared to the ones for the parents or children. Scientific basis has been carefully checked for, avoiding “preconceived ideas” without sufficient evidence.

**Table 1:** Main messages included in the information documents according to the group of intervention

**Topics addressed**	**Nutrition**	**Sustainability**	**Mixed**
**Water**			
Place of water in daily life (thirst, sport)	C/P/T/Po	C/P	C/P/T/Po
“Simplicity” (accessibility) of drinking tap water (practical tips, including taste improvement and bottle washing)	C/P/T	C/P/T/Po	C/P/T/Po
Tap water vs. bottled water for health (minerals)	C/P/T/Po	T	C/P/T
Water origins and safety	C/P/T	C/P/T	C/P/T
Environmental harmful effect of bottled water (from extraction to home)		C/P/T/Po	C/P/T
Limitation of water wastage at home		C/P	P
**Sweetened beverages**			
Recommended frequency of SB consumption	C/P/T/Po	Po	C/P/T/Po
Health harms of sugar	P/T		P/T
Sugar content and acidity of manufactured SB	C/P/T/Po		C/P/T/Po
(Tap) water as a substitution for SB	C/T/Po	C/T/Po	C/T/Po
**Others**			
Tips for changing daily behaviour step by step	C/P/T	P/T	C/P/T
Recipes for healthy and sustainable beverages	P/T/S	P/T/S	P/T/S
Parents/teachers’ modelling and role	P/T	P/T	P/T
Marketing influences	C/P/T/Po	C/P/T	C/P/T/Po
Nutriscore use (tips for choice)	C/P/T	P	C/P/T
Toilet access at school	C/T	T	T
Waste associated with bottled beverages/use of bottle and reusable cups		C/P/T/Po	C/P/T/Po
Cost of bottled beverages	P/T	C/P/T	P/T
Beverages and global warming		C	C

All documents are given on paper and made available on a website, with the access being differentiated according to the group

*C* children leaflet, *P* parent leaflet, *T* pedagogical leaflet for teachers, *Po* posters, *S* summer booklet for children

The difference between the three groups is made by the content in the information provided, all schools (except group #4) being exposed to “water breaks” during the school day, bottles, or cups if they were asked for and meetings. Thus, group #3 receives mixed information related to both nutrition and health issues and sustainability issues. A similar graphic chart is applied to all documents, with specific colours for each group (blue for the nutrition group, green for the sustainability group, and light orange for the mixed group) to enhance the messages.

### Criteria for discontinuing or modifying allocated interventions {11b}

No harmful effect is anticipated for children, so there is no reason to stop the trial by itself or to change allocation. The only change that may occur is the adaptation of messages due to external circumstances.

### Strategies to improve adherence to interventions {11c}

Protocol was developed with school stakeholders. Also, documents were developed with the involvement of the children. For the first leaflets for the children, a pre-test was undertaken in a voluntary school, not included in the trial; their overall perception and understanding of the wording led to the adaptation of the content. Also, the intervention documents have been successively developed, each one considering the feedback potentially received from the precedent document.

### Relevant concomitant care permitted or prohibited during the trial {11d}

All schools, from the intervention groups and the control group, are free to continue their ongoing projects about nutrition and sustainability.

### Provisions for post-trial care {30}

There is no anticipated harm and compensation for trial participation.

### Outcomes {12}

The first quantitative judgement criterion will be the change over time in the mean SB consumption (in ml/day) in the intervention groups compared with the control group (Fig. 1). The secondary criteria of judgement will be the change in the proportion of children consuming SB each day on the one hand and in the mean consumption of water (in ml/day) on the other hand. Quantitative outcomes in ml/day will be evaluated through a 4-day booklet, while the daily consumption will be based on a short frequency questionnaire. These outcomes will be measured at the second wave of data collection and changes estimated by difference with the pre-intervention measurements. Findings at the first post-intervention data collection will also be presented once the trial is finished.

Among the secondary objectives of the DRINK trial, the same outcomes will be used to assess the potential interaction between nutrition- and sustainability-based interventions (Fig. 1). The other three secondary objectives will be assessed using complementary information as described below. Altogether, the information collected and the regular monitoring of the trial development (including cost which is one of the objectives) will also enable to develop a comprehensive process evaluation.

### Participant timeline {13}

**Fig. 3:**
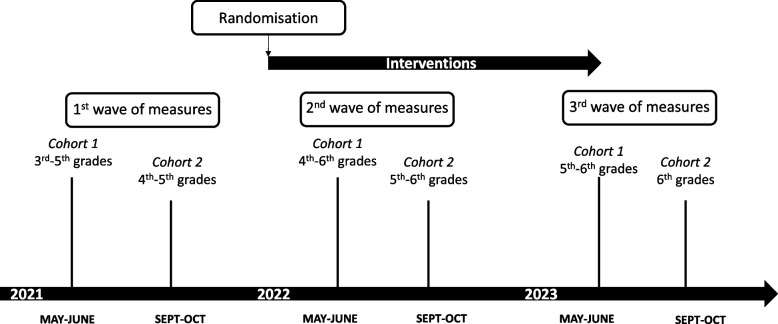
Timeline of the DRINK trial (measurements, randomisation, interventions)

**Fig. 4:**
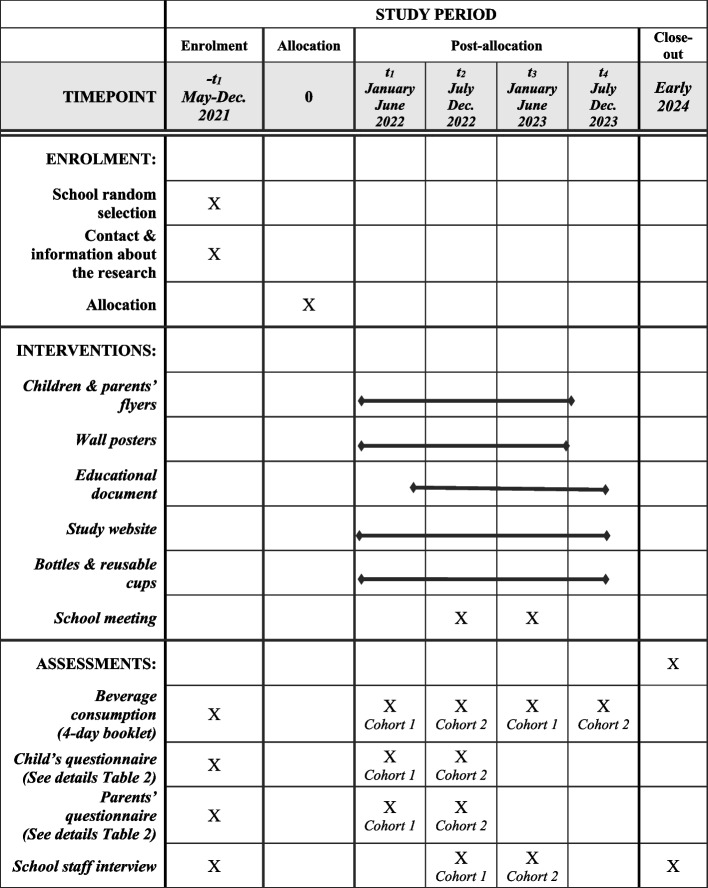
Participant timeline

Despite the COVID-19 impact on schools’ organisation in 2021, the agenda of the research is maintained as initially planned (Figs. 3 and 4). Recruitment and first assessment, prior to randomisation and intervention, were planned to be held in the second period of the school year 2020–2021 for all schools. In essence, they were implemented in May to June 2021 but continued in the beginning of the next school year, due to the lack of availability of some school staff (Fig. 3). Therefore, two cohorts have been formed, including 26 schools and 22 schools, respectively.

For a given child, follow-up will vary depending on his/her grade at entry in the DRINK trial. Children in 3rd and 4th grades (8 to 10 years of age) at inclusion will be followed up during two school years (5th–6th grades at the endpoint, when they are 10 to 12 years old), with an intermediate assessment after 1 year. Those who were schooled in 5th grade at the first evaluation will be followed up for 1 year and will contribute to the intermediate assessment of effectiveness only. Exposure to intervention will be considered as “continuous” during the two school years since actions will have been implemented steadily.

### Sample size {14}

The sample size determination is based on the cluster-randomised factorial plan [45, 46]. The following assumptions have been set for the first criterion of judgement: (i) observed mean of SB consumption, i.e. 200 ml/day (SD: 120 ml) [47]; (ii) a 15% reduction in the intervention group (either nutrition or sustainability), i.e. mean 170 ml/day at the last 2-year follow-up and stability in the control group; (iii) type I error (alpha) of 5%; (iv) statistical power of 80%; and (v) intra-class correlation of 0.08 (estimation based on the “Health Behaviour in School-aged Children” (HBSC) survey carried out in 2018 in French-speaking Belgian primary schools) [48]. The theoretical total number of schools needed is estimated at 48, corresponding to around 3200 students surveyed during the 2 years (based on the same 2018 HBSC survey: on average, four classes in each participating school and 17 students in each of these classes). Since we use a factorial plan, 12 schools are allocated in each group (Fig. 1).

### Recruitment {15}

Random selection of schools to be invited to participate in the trial was based on the official list provided by the Ministry of Education (FWB). In order to ensure a good coverage of the school population, it was stratified by region (Brussels/Wallonia) and by the school socio-economic index (SSEI) stratum (*n* = 3). The SSEI is scored following several characteristics of the families attending the school [48]. We used the tertiles of the SSEI to identify the low, intermediate, and high SSEI of primary schools.

Once they were selected, we contacted the schools to inform them of their selection and to describe the research. Reasons for school refusals were collected (as well as for possible further withdrawal). Among 1379 eligible schools, 168 have been sampled (baseline participation rate: 29%). When they accepted to be part of the research, the first data collection was planned. Ultimately, the six strata of schools are suitably balanced among the participating schools. Furthermore, all school networks are represented.

As a cluster trial, all pupils of the schools in intervention groups receive the intervention. Furthermore, in accordance with the ethical committee advice (see below), no specific recruitment process has been developed for the pupils, except through the letters of information about the data collection.

## Assignment of interventions: allocation

### Sequence generation {16a}

After the first wave of data collection, we assigned the schools to the four groups. All schools were randomly numbered (“rununiform” command in Stata®), and allocation was made by dividing the schools into 4 equal groups. The allocation process was blinded to any information related to schools in an independent database.

### Concealment mechanism {16b}

Given the kind of intervention planned in the DRINK project, further steps cannot be blinded since informing schools and the research team is necessary to achieve the project.

### Implementation {16c}

As soon as the randomisation was made, schools, including those from the control group, were informed of their attributed group.

## Assignment of interventions: blinding

### Who will be blinded {17a}

As an open-label study, it is not possible to blind schools, parents, and children. However, the allocation group is not mentioned in the questionnaires and in the 4-day booklets. During the intervention period, it is also essential for the research team to know the allocation group (for the preparation of information documents, for example). Once the first objective analyses are undertaken, data analysts will be blinded to the allocation group (also see the “Statistical methods for primary and secondary outcomes {20a}” section).

### Procedure for unblinding if needed {17b}

The design is open label with only outcome assessors being blinded so unblinding will not occur.

## Data collection and management

### Plans for assessment and collection of outcomes {18a}

At each wave of evaluation (Fig. 3), we collect information from the school staff, children, and parents. The school staff answer a face-to-face 1-h questionnaire with a dietitian about health promotion actions related to nutrition and sustainability fields including dietary recommendations and food supply, access to tap water, environment-related activities, parents’ involvement in the school activities, and impact of COVID-19 pandemic. In the intervention groups, their perception of the intervention content and organisation is also documented (2nd wave). Repeating such an information collection and completing it with specific questions on their perception about the research at 2nd and 3rd waves will enable to measure the school adherence to the interventions in addition to the participation rates at each evaluation.

Children’s and parents’ self-completed paper questionnaires focus on living conditions, dietary habits, and sustainability components (Table 2). Children complete the questionnaire at school while parents fill it in at home. Some questions come from the HBSC surveys carried out in French-speaking Belgium; therefore, these have been validated elsewhere; others come from validated tools (Table 2). Questions are divided between the two questionnaires depending on the capacity of children to answer them (e.g. parents’ occupation asked in the parents’ questionnaire). A pre-test was undertaken in several classes of a voluntary school (not included in the trial) to improve the wording and simplify some questions, which were too difficult for children of this age.

**Table 2:** Topics^a^ addressed in the questionnaires self-completed by children and parents

	**Children**^**b**^	**Parents**^**c**^
Drink consumption behaviour	Beverage frequency questions (*n* = 6) *Sugar liking* [49] Thirst perception^d^ *Peer influence* [50, 51] Marketing influence [52] Beverage accessibility at home^d^ Tap/bottled water use at home and school^d^	Drinks given for lunchbox, snacks and origin (manufactured, homemade) Home rules for accessibility *Reasons for choosing beverages*
Food consumption behaviour	Short food frequency questionnaire (*n* = 12) Weekly breakfast frequency	Fast-food frequency
Environmental risk perception and behaviour	Behaviour to decrease environmental impact [53, 54]	*Perception of environmental risks* [55] Sensitivity to packaging waste
Health status and behaviour	Self-perceived health status Body weight perception *Tooth brushing*	Declared weight and height^e^ *Sleeping time* *Last dentist visit, previous decay* *Transportation, sport, outside playtime*, *screen duration* Parental perception of physical activity
Demographics: socioeconomic status and conditions of living	School grade, sex, month/year of birth^d^	School grade, sex, month/year of birth
	Family affluence scale	*Parents’ occupation, school attainment*
	*Country of birth*^d^ *Acculturation scale* [56]	*Parents’ country of birth*
	*Siblings*, household composition^d^	
Well-being and social relationship	Satisfaction of life Well-being at school (overall, work, *teachers*) *Friendship* *Perceived self-efficacy* *Relationships with parents*	*Parental role modelling* [57] *Expectation for child’s future* Relationship and* involvement at school* Perception of the school’s role in children’s health^f^

^a^Data in italics are topics addressed only at the 1st wave of data collection

^b^In children, questions come from the HBSC surveys [58] unless another reference is mentioned

^c^In parents, most questions were developed or adapted for the DRINK project unless a reference is mentioned

^d^Children’s questions developed for the DRINK project

^e^Parents are encouraged to measure children

^f^Question asked at wave 2 only

A booklet has been developed and tested for collecting information on beverage consumption. This is a 4-day diary where children use stickers to describe the moment of the day (10 different stickers), type (13), and amount of each beverage consumed (22). They are also encouraged to write information about the characteristics of the beverage (brand and taste) and any additions such as sugar, syrup, or cacao. In 2021, a calibration study was conducted on 114 children to compare declarations, on the same given day, through a 24-h recall administrated by a paediatric dietitian on the phone (considered as the method of reference) and information collected in the booklet. A calibration error will be computed and used at the time of analysis [59].

### Plans to promote participant retention and complete follow-up {18b}

School decision-makers were consulted when the protocol was developed; we collected their opinion mainly about the objectives, the interventions, and the methods to include and follow the schools up. Therefore, research is calibrated to be acceptable for a rather long duration by the school staff, who are frequently requested for various issues, including other studies. Regular email and phone contacts are carried out to enhance the involvement of schools in the project, along with a newsletter describing the progress of the research itself. It is also sent to control group #4 with information on data collection only and without mention of the intervention. The second and third audits at schools include questions about their perception of the research burden, including intervention and organisation for data collection.

### Data management {19}

Daily monitoring is ensured by using tools shared in the research team that help follow up contacts with schools (participation, data collection and intervention material; organisational issues) and to monitor the inclusion and participation at any stage of the project (participating schools and number of questionnaires/4-day booklets received and completed). The school staff manage the list of correspondence between the ID number and names and save an encrypted copy of the list in the server of the School of Public Health (ESP-ULB). Such a list is necessary for the follow-up of each child over time.

When they are sent back to ESP-ULB, parents’ and children’s paper questionnaires are checked for the corresponding ID numbers and for the readability of the written information. They are then sent for a numerical scanning to a specialised firm. A codebook we provided is used by the firm to develop the database containing only numerical codes. Databases are checked for by comparing them with the monitoring tools (ID numbers) and with the codebook (codes’ ranges). Some key frequencies are verified to detect other potential mistakes at the time of scanning. After this first step, outlying values for continuous variables and consistency between some key questions are evaluated. Initially filled in on paper by the dietitians, schools’ audit questionnaires are entered into a form developed using the Limesurvey® software. They include categorical questions which are coded, and free-space texts, which are also entered to facilitate their subsequent analysis.

Lastly, 4-day booklets are entered using an Excel® form specifically developed for the project. A first quality control is made at the time of entry by signalling booklets which will need to be checked one by one for their plausibility and their completion. After the development of the database, the Stata® software is used to recode the inconsistencies (for instance, cacao added to a soup) and to identify extreme amounts of beverage consumption (for each intake and total for each day). All databases are safely stored on the cloud managed by ULB.

### Confidentiality {27}

As described above, the research team has no access to any nominative information. Like any other health workers, researchers are committed to respect confidentiality rules. Children’s and parents’ documents are provided in numbered envelopes that they can keep throughout the data collection period, so that school staff cannot read answers to questionnaires and 4-day booklets. Furthermore, teachers are explicitly encouraged to respect the confidentiality of answers when children complete the self-administrated questionnaire in class and when they gather the 4-day booklets before sending them to the research team.

### Plans for collection, laboratory evaluation, and storage of biological specimens for genetic or molecular analysis in this trial/future use {33}

No biological specimens are collected (also see the “Additional consent provisions for collection and use of participant data and biological specimens {26b}” section).

## Statistical methods

### Statistical methods for primary and secondary outcomes {20a}

After presenting the characteristics of each group at baseline, statistical analysis will include assessment of the primary and secondary criteria of judgement and secondary objective. Intention-to-treat analyses will be carried out. At the time of statistical analyses, the group allocation will be blinded in the database.

For the effectiveness criteria of judgement, multilevel regression analyses will be used to consider the randomisation by cluster (and not by individual) since the number of clusters is rather high (48 schools) [60]. Differences in the beverage consumption (in ml/day, from the 4-day diary) between the endpoint and the initial assessment (undertaken before trial allocation of schools) will be computed for each child followed up over this period, such values being the outcome to evaluate. We will also compare the proportions of children drinking SB each day based on the frequency questions. After analysing the 2-year post-intervention vs. initial data, we will carry out the same analyses at 1-year post-intervention among (1) all children followed up for 2 years and (2) children with data available at inclusion and at 1-year post-intervention. This complementary assessment will contribute to the understanding of the trial effectiveness, if any.

### Interim analyses {21b}

No interim analysis is planned in accordance with the section {20a}.

### Methods for additional analyses (e.g. subgroup analyses) {20b}

Regressions will be adjusted for various characteristics related to beverage consumption in children and parents, and differences between schools at randomisation. Adjustment variables will be chosen based on the differences observed between the randomised groups at inclusion. Stratified analyses are planned based on the inclusion characteristics already chosen: child body weight status, family socio-economic status, and some school indicators such as SSEI and province. Purposely, a predefined and limited number of stratified analyses will be undertaken, and repetition of analysis will be corrected for.

### Methods in analysis to handle protocol non-adherence and any statistical methods to handle missing data {20c}

Adherence to the intervention will be described based on the school audits at times 2 and 3, to better understand the findings, but will not be taken into account in the analyses of effectiveness. In addition, missing information for children and parents will be addressed through sensitivity analysis by using adapted statistical methods such as a two-stage targeted minimum loss-based estimator [61].

### Plans to give access to the full protocol, participant-level data, and statistical code {31c}

The protocol registered will be updated and made available under request for documents which are not available on the ISRCTN website. Intervention documents, information letters and questionnaires are available upon request. Furthermore, the ULB is currently developing a plan for data deposition of research for the coming years; we will follow the institutional recommendations as soon as rules, procedures, and security are established. In the meantime, databases will be stored and protected in the cloud of the ULB IT system, and statistical coding will be made available if requested by reviewers for instance. Scientific collaboration will be welcomed once the main objectives have been analysed.

## Oversight and monitoring

### Composition of the coordinating centre and trial steering committee {5d}

The coordinating centre comprises the principal investigator, a project leader and a part-time researcher who are supported by the paediatric dietitians and administrative/logistic staff. Regular meetings are organised to follow up the study progress and schools’ participation, to plan the next steps for intervention implementation and data collection, and to address any question related to data quality.

An international scientific committee has been established to support the development of the protocol, data collection, and intervention content and to follow the progress of the research. It is made up of epidemiologists, psychologists, public health researchers, and one economist, all having previous experience in one or several fields covered in the DRINK project (see the “Acknowledgments” section). Three meetings were organised before the beginning of the project, and yearly meetings are planned during the whole length of the project. In addition, a steering committee gathers stakeholders and education authorities in Belgium. It is informed about the progress of the research and may give feedback on the general context, but members do not interfere with the scientific content of the research.

### Composition of the data monitoring committee, its role, and reporting structure {21a}

Considering no specific harmful effect is expected to lead to the trial stopping, a data safety monitoring board has not been considered as necessary.

### Adverse event reporting and harms {22}

No adverse event or harmful effect even minor is anticipated.

### Frequency and plans for auditing trial conduct {23}

During the protocol preparation (2020–2021), the Scientific committee met three times. Since then, the meetings occur once a year. The steering committee also meets once a year. For both committees, a comprehensive update of the trial is presented, and any concern is fully addressed. The research team meets every 2 weeks to discuss the progress of the trial as well as any issues that may occur.

### Plans for communicating important protocol amendments to relevant parties (e.g. trial participants, ethical committees) {25}

Substantial amendments to the protocol will be notified to the Ethical Committee and the International Scientific Committee for approbation (for instance, the split into two cohorts (see the section {13}) has been approved by the Scientific Committee). If amendments would affect children, parents, and school staff, they will also be informed. Non-substantial amendments will not be communicated but related changes will be documented and detailed in the further publications.

## Dissemination plans {31a}

Dissemination of results will include several components. Obviously, we will present our findings in various peer-reviewed journals (following the CONSORT extension for cluster randomised trials) and at international conferences. Recommendations from the International Committee of Medical Journal Editors will be followed to define authorship eligibility and order. Furthermore, our regional involvement in health promotion programmes in Brussels and Wallonia will be used to extend the result dissemination to various stakeholders and actors of the field in these domains (education staff, health promotion at school, nutrition, environment). The staff at the participating schools will be invited to a specific presentation of the results. This will be an opportunity to collect their final feedback on all the components of our research. Finally, our research has been developed so that interventions could be generalised. Therefore, we will present our findings, whether positive, null, or negative, to the decision-makers of health promotion at school (including the Ministry of Health, FWB). Indeed, beyond our own research, the evaluation of health promotion interventions needs to be developed, independently of the topics addressed.

## Discussion

Careful evaluation of health promotion at school is crucial. Indeed, school is identified as an interesting health-related intervention setting, but efficiency of health promotion at school has not been sufficiently evaluated so far. Several systematic reviews underlined the methodological limitations of the relatively few published studies. Previous studies lacked several methodological components, that will be addressed in the DRINK protocol. For instance, the theoretical sample size leads to a number of schools much higher than what has often been published, not due to an expected small effect size, but because it has been computed by taking into account the cluster design. Another strength is the long-term evaluation. On the one hand, it will enable to measure the behavioural changes that are expected to last for a long time. On the other hand, the long-term follow-up may decrease school participation and increase the children’s contamination if some of them change schools during the trial. This is why we have measured the burden of intervention and of data collection. However, the school staff must still organise the distribution of the documents and keep the list of correspondence between the names and ID numbers.

Multiform interventions aimed at being included in the daily life of schools have purposely been adapted to the school context. Long-term school adherence is a key component of such interventions, while staff are frequently requested either for research projects or for the inclusion of actions beyond their main attributions. Therefore, interventions are not fully standardised between schools of a given group, but at the same time, such an adaptation leads to a more realistic implementation. Indeed, when the research was developed, concern for a future generalisation was kept in mind continuously. As mentioned in the “Introduction” section, the originality of our research lies in the factorial randomisation plan aiming at evaluating the effectiveness of two series of promoters towards a healthy diet; health and nutrition, environmental sustainability, or combined. This research protocol and its further findings will open new opportunities for research in health promotion, especially regarding dietary behaviours.

The measurement tools (questionnaires and 4-day booklets) have also been carefully developed leading to a comprehensive evaluation of the expected effectiveness of interventions. The 4-day booklet, including coloured stickers, is really appreciated by children so far, and the calibration study will enable the measure of error against a 24-h recall made by trained dietitians. Given the age of the children, it is necessary to ask parents to answer some questions through a questionnaire in addition to theirs (Table 2). Though this complexifies the data collection, measuring their attitude toward beverages will complete the assessment of familial facilitators and barriers in the children’s dietary behaviour changes. A similar purpose is addressed through the interview of the school staff: information collected will inform the feasibility of future generalisation. Despite regular contacts made by the research team, the main concern is the willingness of school staff to organise the data collection, especially in the control group. The completion rate of questionnaires and 4-day booklets is expected to be lower than if research interviews had been undertaken; however, this method is too costly in the frame of our grants. Lastly, the COVID-19 pandemic has widely affected schools and their staff, making the implementation of such research projects in 2021 and 2022 difficult. School participation rate will therefore be underestimated compared to more usual conditions.

Developing and evaluating health promotion actions related to healthy and sustainable diet offer important perspectives for public health. To date, combining and assessing the possible interaction between nutrition-based and sustainability-based interventions has not been studied yet. Our research will provide an original and topical insight of health promotion at school around dietary behaviours. The dissemination plan will enable to widely inform stakeholders, school staff, and families, in addition to the scientific community through the usual medium (articles, conferences), about the research findings in 2023–2025.

## Trial status

The first wave of data collection took place in Spring and Autumn 2021, and the second wave was just finished at the beginning of 2023. The third and last wave of data collection is planned for the period from April to December 2023. The main results will be disseminated by the end of 2024, through publications and conference communications.

## Data Availability

Data collected in the frame of the DRINK trial will be made available in the future for collaborative research questions once the main objectives have been analysed.

## Abbreviations

cRCT: Cluster randomised controlled trial
DRINK: Decreasing consumption of sugar-sweetened beverages and Raising tap water consumption through Interventions based on Nutrition and sustainability for Kids
ESP: *Ecole** de Santé **Publique* (School of Public Health)
FWB: Federation of Wallonia-Brussels
HBSC: Health Behaviour in School-aged Children
SB: Sweetened beverages
SSEI: School socio-economic index
TPB: Theory of planned behaviour
ULB: *Université libre de Bruxelles*
